# Effect of COVID‐19 vaccine on menstrual experience among females in six Arab countries: A cross sectional study

**DOI:** 10.1111/irv.13088

**Published:** 2022-12-28

**Authors:** Sajeda Ghassan Matar, Anas Zakarya Nourelden, Ahmed Assar, Eshak I. Bahbah, Areej M. Alfryjat, Elfatih A. Hasabo, Suzan A. Matar, Shatha Nizar Bishtawi, Mays Alhoubani, Ahmad Bassam Yahia, Khaled Mohamed Ragab, Lina Mohammad Salameh, Lana Saif Eddin Salameh, Mohamed Sayed Zaazouee, Mohammed Al‐kafarna, Alaa Ahmed Elshanbary, Hossam Waleed Almadhoon, Shahed Toulaq Bakdash, Ola Awad Babiker Adam, Abdelkader Nabeel Malih, Shimaa Abo elfotoh Habash, Rakia Mohamed Taha Basiouny, Afaf Ahmad, Raghda Mohammed Ahmed Hamid, Balsam Younan Habib, Dalia Nasr Elokl, Hiba Hatim Abdalraheem, Esraa Adel Atia, Nazik Ibrahim Ahmed Yousif, Fida Hussien Al‐Ali, Israa Mohammed Alshaer, Fatima Elsidieg Abdulali, Hadil Abu Ayesh, Anwar Yousef Jabari, Raneem Ahmed Egzait, Nameer Amer Abu Munshar, Aseel Ahmad Alkhraibat, Aisha Hasan ibreerah, Iman A. Basheti

**Affiliations:** ^1^ International Medical Research Association (IMedRA) Cairo Egypt; ^2^ School of Pharmacy Applied Science Private University Amman Jordan; ^3^ Faculty of Medicine Al‐Azhar University Cairo Egypt; ^4^ Faculty of Medicine Menoufia University Shebin El‐Kom Egypt; ^5^ Faculty of Medicine Al‐Azhar University Damietta Egypt; ^6^ Gynecology Department Jordanian Ministry of Health Amman Jordan; ^7^ Department of Anatomy, Faculty of Medicine University of Khartoum Khartoum Sudan; ^8^ Department of Clinical Laboratory Sciences The University of Jordan, School of Science Amman Jordan; ^9^ Faculty of Medicine The University of Jordan Amman Jordan; ^10^ Faculty of Pharmacy The University of Jordan Amman Jordan; ^11^ Faculty of Medicine Hashemite University Amman Jordan; ^12^ Faculty of Medicine Minia University Minia Egypt; ^13^ Faculty of Medicine Al‐Azhar University Assuit Egypt; ^14^ Faculty of Pharmacy Al‐Azhar University – Gaza Gaza Strip Palestine; ^15^ Faculty of Medicine Alexandria University Alexandria Egypt; ^16^ Institute of Biodiversity, One Health and Veterinary Medicine University of Glasgow Glasgow UK; ^17^ Faculty of Medicine Kalamoon University Rif‐Dimashq Syria; ^18^ Faculty of Medicine Al‐Neelain University Khartoum Sudan; ^19^ Faculty of Medicine Tanta University Tanta Egypt; ^20^ Faculty of Medicine October 6 University Giza Egypt; ^21^ Faculty of Medicine Damascus University Damascus Syria; ^22^ Faculty of Pharmacy University of Khartoum Khartoum Sudan; ^23^ Faculty of Medicine Tartus University Tartus City Syria; ^24^ Faculty of Pharmacy Al‐Neelain University Khartoum Sudan; ^25^ Faculty of Medicine University of Khartoum Khartoum Sudan; ^26^ Faculty of Medicine Palestine Polytechnic University‐ Hebron West Bank Palestine; ^27^ Faculty of Medicine Tripoli University Tripoli Libya; ^28^ Faculty of Medicine Misurata University Misurata Libya; ^29^ Faculty of Medicine Al‐Azhar University – Gaza Gaza Strip Palestine; ^30^ Faculty of Pharmacy Jordan University of Science and Technology Irbid Jordan; ^31^ Department of Clinical Pharmacy and Therapeutics Faculty of Pharmacy Applied Science Private University Amman Jordan; ^32^ Faculty of Pharmacy The University of Sydney Sydney Australia

**Keywords:** COVID‐19, cross‐sectional, menstrual health, menstruation, vaccine, women's health

## Abstract

**Background:**

There have been varying reports on the potential occurrence and severity of changes to menstruation including the median cycle length, days of bleeding, bleeding heaviness, and menstrual pain, following receipt of COVID‐19 vaccinations. We aimed to assess potential postvaccination menstrual changes in women residing in the Middle East.

**Methods:**

We implemented a cross‐sectional online survey‐based study. Data about the participants' demographic characteristics, menstruation experience, and vaccination status were collected and analyzed among six Arab countries.

**Results:**

Among 4942 menstruating females included in this study, females who had received one or more doses of COVID‐19 vaccination reported a higher frequency of back pain, nausea, tiredness, pelvic pain with periods, unprescribed analgesics use, and passage of loose stools. They also reported higher scores describing average and worst menstrual pain. Fully vaccinated females reported heavier flow and more days of bleeding.

**Conclusion:**

Our findings indicate that COVID‐19 vaccine may have an effect on menstruation in terms of menstrual pain and bleeding heaviness. The evidence needs to be further investigated in longitudinal studies.

## 1 INTRODUCTION

After the spread of the new coronavirus by the end of 2019,^1^ many pharmaceutical companies worked on developing a vaccine for COVID‐19, but only a few have successfully released vaccines that later were distributed worldwide. Vaccine development was claimed to help limit the spread of the virus, prevent death, and decrease hospitalization. Multiple technology platforms were applied to develop vaccines, including mRNA vaccines, viral vector vaccines, and inactivated vaccines.^2^ Emergency approvals were provided for those vaccines to limit the spread of the coronavirus and reduce its impact. While these vaccines proved to be safe for human use in the short term, some side effects were associated with their administration including pain at the site of injection, swelling, and redness, as well as fatigue, chills, fever, myalgia, headache, and nausea.^3^ On the other hand, several reports of thromboembolic events in subjects who had been administered Vaxzevria were reported^4^ as well as rare side effects associated with Pfizer‐BioNTech vaccines such as Bell's palsy and lymph node swelling and tenderness.^5^ Sputnik vaccine was associated with side effects that were not common, such as a temporary increase in liver enzymes such as serum creatinine and CPK, a decrease in neutrophils, an increase in lymphocytes, and either an increase or decrease in platelets.^6^ Some cases of thrombotic thrombocytopenia were reported after the administration of the Johnson and Johnson vaccine.^7^

A few studies have raised the possibility of menstrual changes after receipt of COVID‐19 vaccination.^8^, ^9^ To alleviate concerns about the safety of these vaccines among menstruating females, this study was designed to assess the severity of post‐vaccination changes to menstruation including the median cycle length, days of bleeding, bleeding heaviness, and menstrual pain.

## 2 METHODS

### 2.1 Study design and setting

A multicenter cross‐sectional study was conducted using a self‐administered pre‐piloted anonymous questionnaire (Supporting Information S1). The study was conducted according to the STROBE guidelines for reporting and conducting cross‐sectional studies.^10^ Females from six Arab countries were invited to participate in this study; females who did not receive any dose of the COVID‐19 vaccine were considered the control group.

The study was conducted online by distributing the questionnaire among females via social media platforms in six Arab countries including Jordan, Palestine, Syria, Egypt, Sudan, and Libya.

### 2.2 Inclusion and exclusion criteria

We included menstruating female participants above 18 years of age, and we excluded females who were pregnant, breastfeeding, taking oral contraceptives or any other hormonal therapy, using intrauterine devices, or those who had endometriosis or polycystic ovarian syndrome.

### 2.3 Study instruments

Questions about the following information were included:

1. *1‐Demographic Characteristics*: Females were invited to answer questions regarding their age, country, residency, education, work status, weight, height, smoking status, exercise, and exposure to stressful conditions.
2. *2‐Menstruation Experience*: The Women's Health Symptoms Survey (WHSS)^11^ questionnaire was used to assess women's answers regarding their pain during menstruation and if it had an impact on their physical activities, such as going to work or doing sports, or if they developed thigh, anal and/or back pains, and dysuria. Questions about severity, frequency of pelvic pain, and analgesic use were included, and pain score associated with menstruation was scored on average and in worst cases. Furthermore, questions regarding bleeding heaviness, duration of menstruation, the number of days between the start of one period and the beginning of the next one were enclosed as well. Five questions were added to cover other aspects such as bowel movements associated with pain and the frequency of defecation and the texture of the stool during menses.
3. *3‐COVID‐19 Vaccine Administration Data*: All participants who administered the COVID‐19 vaccine were asked about the type, the number of doses, and the time period since they received the vaccine.
4. *4‐History of COVID‐19 infection Data*: All participants who had a history of COVID‐19 infection were asked about the severity of their infection, assessed by the requirement of oxygen therapy, presence of pneumonia, requirement of any type of ventilation, admission to hospital status, and duration since they got COVID‐19 infection.

### 2.4 Sampling and sample size calculation

A convenience sampling method was used to acquire online responses from the participants to the e‐survey questionnaire. The sample size was calculated as two independent female samples from each country; one for the control group for females who did not receive the COVID‐19 vaccine, and the second for females who previously received one dose or more of the COVID‐19 vaccine. The equation *n* = *z* ^2^ *P(1 − P)/d* ^2^ was used with 95% CI, 50% response distribution, and 0.05 margin of error.^1^ A sample of 384 participants was considered a minimal sample for each group in each country.

### 2.5 Data collection and handling

Data collection started in November 2021 and ended in December 2021 by online distributed questionnaires on different internet platforms in the six participating Arab countries. Voluntary participation and comprehension of informed consent among females were set and confidentiality was ensured through proper data management and security.

### 2.6 Ethical considerations

Ethical approval of research (No. 2022‐PHA‐1) was obtained from the Institutional Review Board (IRB) of the Applied Science Private University (ASU), Jordan.

### 2.7 Statistical analysis

Descriptive analyses were conducted (frequency and percentage) to describe demographic characteristics, COVID‐19 vaccine administration data, COVID‐19 infection data, and menstruation experience and symptoms. A Chi‐square test was used to explore the relationship between menstruation experience and vaccination status, and to compare the different types of vaccines in association with the reported menstrual experience. Mann–Whitney *U* test was used to compare pain scores with the vaccination status.

Logistic regression was used to assess the association of vaccination with menstrual experience outcomes adjusting for the demographics. Three models were employed in the data analysis, the first included demographic characteristics that are known to not affect the menstrual experience (country, residency, education, and work), whereas the second included demographics that potentially affect the menstrual experience (smoking, stressful conditions, entertainment sports, COVID‐19 infection, BMI, age), and the third model included all the demographic characteristics. A p‐value of less than 0.05 was considered significant.

## 3 RESULTS

A total of 6454 females participated in this study, although 1512 were excluded since they did not meet the inclusion criteria. Among 4942 female participants who were included from six Arab countries with a mean age of 24.02 (SD = 5.73) were included in the study. The mean Body Mass Index (BMI) of the sample was 23.56 (SD = 4.79), 84.6% of them had a college degree or above, and 2919 (59.1%) of them were vaccinated against COVID‐19. The most common types of vaccine received were Pfizer (27.2%), Sinopharm (24.7%) or Sinovac, and AstraZeneca (24.3%). Full demographic characteristics can be found in Table 1.

**TABLE 1:** Demographic characteristics

Basic characteristics	Number (%)^a^	Mean (SD)
**Total number of samples = 4942**		
Age, years		24.02 (5.73)
Body mass index (BMI)		23.56 (4.79)
Country		
Jordan	677 (13.7%)	
Syria	825 (16.7%)	
Palestine	908 (18.4%)	
Egypt	899 (18.2%)	
Libya	775 (15.7%)	
Sudan	856 (17.3%)	
Residency		
Urban	3822 (77.3%)	
Rural	1120 (22.7%)	
Education		
Below college	760 (15.4%)	
College and above	4182 (84.6%)	
Work		
Unemployed	3438 (69.6%)	
Part‐time	753 (15.2%)	
Full‐time	751 (15.2%)	
Smoking		
Currently smoker	252 (5.1%)	
Not at all	4617 (93.4%)	
Ex‐smoker	73 (1.5%)	
**Total number of participants who received COVID‐19 vaccine = 2919**		
Vaccinated		
Yes	2919 (59.1%)	
No	2023 (40.9%)	
Type of vaccine		
AstraZeneca	709 (24.3%)	
Johnson and Johnson	134 (4.6%)	
Moderna	51 (1.7%)	
Pfizer	794 (27.2%)	
Sinopharm or Sinovac	722 (24.7%)	
Sputnik	509 (17.4%)	
Doses received		
First dose only	991 (33.9%)	
Two doses	1747 (59.8%)	
Two doses and third additional	36 (1.2%)	
One dose of Johnson and Johnson	145 (5.0%)	
Time since first dose, months		5.7 (3.44%)
Time since second dose, months		4.3 (2.83%)
**Total number of participants who have a history of COVID‐19 infection = 1838**		
COVID infected		
Yes	1838 (48.7%)	
No	1935 (51.3%)	
Time since infection, months		7.32 (4.67)
Symptoms		
No symptoms	154 (8.4%)	
Mild (cough, muscle and joint pain, loss of smell or taste sensations%)	1335 (72.6%)	
More severe symptoms	349 (19%)	
O_2_ saturation below 90%		
Yes	152 (9.3%)	
No	1048 (57%)	
Not sure	638 (34.7%)	
Pneumonia		
Yes	117 (6.4%)	
No	1470 (80%)	
Not sure	251 (13.7%)	
O_2_ therapy		
Yes	83 (4.5%)	
No	1735 (94.4%)	
Not sure	20 (1.1%)	
Ventilator requirement		
Yes	39 (2.1%)	
No	1786 (97.2%)	
Not sure	13 (0.7%)	
Place of isolation during infection		
Home	1790 (97.4%)	
Hospital	44 (2.4%)	
ICU	4 (0.2%)	
**Total number of participants in this section = 4942**		
Age of Menarche, years		13.25 (1.58)
Menstruation regularity		
Yes	3668 (74.2%)	
No	651 (13.2%)	
Not sure	623 (12.6%)	
Mentally or physically stressful situations in 3 months		
Yes	2462 (49.8%)	
No	1212 (24.5%)	
Not sure	1268 (25.7%)	
Sports practice in 3 months		
Yes	1542 (31.2%)	
No	3400 (68.8%)	
Frequency of such sports practice		
No	2499 (50.6%)	
Sometimes	890 (18%)	
Regularly	248 (5%)	
Frequently	471 (9.5%)	
Daily	91 (1.8%)	
Not sure	743 (15%)	
Cessation of sports practice due to pelvic pain		
Yes	616 (19%)	
No	2622 (81%)	
Cessation of sports practice due to menstruation		
Yes	1435 (40.8%)	
No	2083 (59.2%)	

^a^ Valid percent.

Only 1838 (48.7%) participants reported a history of COVID‐19 infection, nearly two thirds of them (72.6%) suffered from mild symptoms only (cough, muscle, and joint pain, loss of smell or taste sensations), while 152 (9.3%) had oxygen saturation below 90%. Minority (4.5%) received oxygen therapy, and (2.1%) required a ventilator. Most of the participants were isolated at home (97.4%). Full COVID‐19 related characteristics can be found in Table 1.

The mean age for menarche was 13.25 (SD = 1.58) years and nearly two‐thirds of the participants (74.2%) had regular cycles. Most of the participants (74.2%) experienced stressful situations, mentally or physically, during the 3 months before participation in the current study, and 1542 (31.2%) practiced sports activity during the past 3 months. Full data can be found in Table 1.

Participants who received one or more dose of COVID‐19 vaccine had a significantly higher frequency of pelvic pain (84.5%) than the unvaccinated participants (81.6%, *p* = 0.006). Similar outcomes were reported for back pain experienced by vaccinated participants (82.9%) versus non‐vaccinated participants (77.9%, *p* < 0.001), thigh pain (63.9% vs. 61%, *p* = 0.045), nausea (43% vs. 40%, *p* = 0.036), tiredness (89.7% vs. 87.1%, *p* = 0.005), pelvic pain (85.6% vs. 81.9%, *p* < 0.001), and taking pain‐killers for the pain without prescription (62.7% vs. 57.2%, *p* < 0.001). Average menstrual pain and worst menstrual pain scores were reported significantly more frequently in the vaccinated group than the unvaccinated. Full comparison can be found in Table 2.

**TABLE 2:** Comparing vaccinated (either fully or partially%) and not vaccinated using chi‐square

Symptoms in the last 3 months	Vaccinated *N* (%)^a^	Not vaccinated at all *N* (%)^a^	*p* value
How heavy is your menstrual flow			
Mild	245 (8.4%)	163 (8.1%)	0.09
Moderate	1951 (66.8%)	1326 (65.5%)	
Heavy	635 (21.8%)	446 (22%)	
Cannot remember	88 (3%)	88 (4.3%)	
How many days are there between the start of one period and the start of the next on average?			
Less than 21 days	151 (5.2%)	99 (4.9%)	0.461
22–24 days	573 (19.6%)	407 (20.1%)	
25–28 days	1171 (40.1%)	834 (41.2%)	
29–32 days	617 (21.1%)	384 (19%)	
33–35 days	146 (5%)	118 (5.8%)	
More than 36 days	56 (1.9%)	33 (1.6%)	
Cannot determine due to irregularity	205 (7%)	148 (7.3%)	
Do you have Pelvic pain			
Yes	2467 (84.5%)	1650 (81.6%)	**0.006**
No	452 (15.5%)	373 (18.4%)	
Do you have anal pain			
Yes	611 (20.9%)	382 (81.1%)	0.077
No	2308 (79.1%)	1641 (18.9%)	
Do you have anal bleeding			
Yes	252 (8.6%)	161 (8%)	0.399
No	2667 (91.4%)	1862 (92%)	
Do you have pain at urination			
Yes	426 (14.6%)	273 (13.5%)	0.276
No	2493 (85.4%)	1750 (86.5%)	
Do you have bleeding at urination			
Yes	822 (28.2%)	533 (26.3%)	0.16
No	2097 (71.8%)	1490 (73.7%)	
Do you have back pain			
Yes	2421 (82.9%)	1576 (77.9%)	**>0.001**
No	498 (17.1%)	447 (22.1%)	
Do you have thigh pain			
Yes	1864 (63.9%)	1235 (61%)	**0.045**
No	1055 (36.1%)	788 (39%)	
Do you have nausea			
Yes	1256 (43%)	810 (40%)	**0.036**
No	1663 (57%)	1213 (60%)	
Do you have tiredness			
Yes	2617 (89.7%)	1762 (87.1%)	**0.005**
No	302 (10.3%)	261 (12.9%)	
Period related pelvic pain in past 3 months			
Yes	2499 (85.6%)	1657 (81.9%)	**>0.001**
No	420 (14.4%)	366 (18.1%)	
Frequency of period related pelvic pain in past 3 months			
Sometimes	583 (22.6%)	477 (2704%)	**>0.001**
Usually	562 (21.8%)	430 (2407%)	
Always	1437 (55.7%)	831 (47.8%)	
Administration of unprescribed pain killers			
Yes	1677 (62.7%)	1036 (57.2%)	**>0.001**
No	997 (37.3%)	774 (42.8%)	
Administration of prescribed pain killers			
Yes	307 (11.6%)	207 (11.4%)	0.912
No	2351 (88.4%)	1602 (88.6%)	
In the last 3 months, has your period pain prevented you from going to work or carrying out your daily activities (even if taking pain‐killers%)?			
Occasionally (with 1 in 3 of my periods%)	896 (33.5%)	576 (31.8%)	0.42
Often (with 2 in 3 of my periods%)	343 (12.8%)	221 (12.2%)	
Always (with every period)	288 (10.8%)	192 (10.6%)	
Never	1149 (42.9%)	823 (45.4%)	
In the last 3 months, have you had to lie down for any part of the day or longer because of your period pain?			
Occasionally (with 1 in 3 of my periods%)	813 (30.3%)	588 (32.4%)	0.353
Often (with 2 in 3 of my periods%)	594 (22.1%)	381 (21%)	
Always (with every period)	956 (35.6%)	619 (34.1%)	
Never	320 (11.9%)	228 (12.6%)	
When you had period pain in the last 3 months how often did this pain get better or stop after you had a bowel movement?			
Always	49 (1.7%)	30 (1.5%)	0.423
Most of the times	203 (7%)	161 (8%)	
Often	418 (14.3%)	272 (13.4%)	
Sometimes	1109 (38%)	740 (36.6%)	
Rarely or never	1140 (39.1%)	820 (40.5%)	
When you had period pain in the last 3 months how often did you have more frequent bowel movements?			
Always	32 (1.1%)	18 (0.9%)	0.812
Most of the times	153 (5.2%)	95 (4.7%)	
Often	333 (11.4%)	241 (11.9%)	
Sometimes	1008 (34.5%)	709 (35%)	
Rarely or never	1393 (47.7%)	960 (47.5%)	
When you had period pain in the last 3 months how often did you have less frequent bowel movements?			
Always	101 (3.5%)	41 (2%)	**0.002**
Most of the times	305 (10.4%)	174 (8.6%)	
Often	489 (16.8%)	359 (17.7%)	
Sometimes	955 (32.7%)	645 (31.9%)	
Rarely or never	1069 (36.6%)	804 (39.7%)	
When you had period pain in the last 3 months were your stools (bowel movements%) looser?			
Always	134 (4.6%)	67 (3.3%)	**0.012**
Most of the times	389 (13.3%)	237 (11.7%)	
Often	541 (18.5%)	341 (16.9%)	
Sometimes	772 (26.4%)	573 (28.3%)	
Rarely or never	1083 (37.1%)	805 (39.8%)	
When you had period pain in the last 3 months were your stools (bowel movements%) harder?			
Always	33 (1.1%)	31 (1.5%)	0.798
Most of the times	159 (5.4%)	107 (5.3%)	
Often	307 (10.5%)	211 (10.4%)	
Sometimes	785 (26.9%)	551 (27.2%)	
Rarely or never	1635 (56%)	1123 (55.5%)	
Pain score as average (mean, SD)	5.8 (2.33)	5.53 (2.41)	>0.001
Pain score at worst cases (mean, SD)	6.97 (2.33)	6.68 (2.38)	>0.001
Days of bleeding (mean, SD)	5.7 (2.06)	5.66 (1.84)	0.76

*Note*: Statistically significant values are presented in bold.

^a^ Valid percent.

Participants who were fully vaccinated (received two doses or one dose of Johnson and Johnson for 3 months or more) were more likely to experience back pain (82.3%) when compared to unvaccinated participants (77.9%, *p* = 0.004). The frequency of all of the following was also higher in the fully vaccinated group compared to the non‐vaccinated; nausea (44.2% vs. 40%, *p* = 0.024), tiredness (90.5% vs. 87.1%, *p* = 0.004), pelvic pain with periods (85.3% vs. 81.9%, *p* = 0.013), taking pain‐killers for the pain without prescription (65.1% vs. 57.2%, *p* < 0.001), the fully vaccinated participants also had higher average and worst pain scores and significantly more days of bleeding. Full comparisons are shown in Table 3.

**TABLE 3:** Comparing fully vaccinated and not vaccinated people

Symptoms in the last 3 months	Fully vaccinated	Not vaccinated at all	*p* value
	*N* (%)^a^	*N* (%)^a^	
How heavy is your menstrual flow usually			
Mild	109 (9.6%)	163 (8.1%)	**0.04**
Moderate	716 (62.9%)	1326 (65.5%)	
Heavy	280 (24.6%)	446 (22%)	
Cannot remember	34 (3%)	88 (4.3%)	
How many days are there between the start of one period and the start of the next on average?			
Less than 21 days	48 (4.2%)	99 (4.9%)	0.881
22–24 days	225 (19.8%)	407 (20.1%)	
25–28 days	482 (42.3%)	834 (41.2%)	
29–32 days	230 (20.2%)	384 (19%)	
33–35 days	60 (5.3%)	118 (5.8%)	
More than 36 days	18 (1.6%)	33 (1.6%)	
Cannot determine due to irregularity	76 (6.7%)	148 (7.3%)	
Do you have pelvic pain			
Yes	954 (83.8%)	1650 (81.6%)	0.12
No	185 (16.2%)	373 (18.4%)	
Do you have anal pain			
Yes	242 (21.2%)	382 (18.9%)	0.109
No	897 (78.8%)	1641 (81.1%)	
Do you have anal bleeding			
Yes	111 (9.7%)	161 (8%)	0.085
No	1028 (90.3%)	1862 (92%)	
Do you have pain at urination			
Yes	167 (14.7%)	273 (13.5%)	0.363
No	972 (85.3%)	1750 (86.5%)	
Do you have bleeding at urination			
Yes	306 (26.9%)	533 (26.3%)	0.751
No	833 (73.1%)	1490 (73.7%)	
Do you have back pain			
Yes	937 (82.3%)	1576 (77.9%)	**0.004**
No	202 (17.7%)	447 (22.1%)	
Do you have thigh pain			
Yes	726 (63.7%)	1235 (61%)	0.134
No	413 (36.3%)	788 (39%)	
Do you have nausea			
Yes	503 (44.2%)	810 (40%)	**0.024**
No	636 (55.8%)	1213 (60%)	
Do you have tiredness			
Yes	1031 (90.5%)	1762 (87.1%)	**0.004**
No	108 (9.5%)	261 (12.9%)	
Period related pelvic pain in past 3 months			
Yes	972 (85.3%)	1657 (81.9%)	**0.013**
No	167 (14.7%)	366 (18.1%)	
Frequency of period's related pelvic pain in past 3 months			
Sometimes	211 (21%)	477 (27.4%)	**>0.001**
Usually	222 (22.1%)	430 (24.7%)	
Always	571 (56.9%)	831 (63.4%)	
Administration of unprescribed analgesics for period pain			
Yes	684 (65.1%)	1036 (57.2%)	**>0.001**
No	366 (34.9%)	774 (42.8%)	
Administration of prescribed analgesics for period pain			
Yes	118 (11.3%)	207 (11.4%)	0.917
No	925 (88.7%)	1607 (88.6%)	
In the last 3 months, has your period pain prevented you from going to work or carrying out your daily activities (even if taking pain‐killers%)?			
Occasionally (with 1 in 3 of my periods%)	342 (32.5%)	576 (31.8%)	0.935
Often (with 2 in 3 of my periods%)	121 (11.5%)	221 (12.2%)	
Always (with every period)	114 (10.8%)	192 (10.6%)	
Never	475 (45.2%)	823 (45.4%)	
In the last 3 months, have you had to lie down for any part of the day or longer because of your period pain?			
Occasionally (with 1 in 3 of my periods%)	312 (29.6%)	588 (32.4%)	0.395
Often (with 2 in 3 of my periods%)	223 (21.2%)	381 (21%)	
Always (with every period)	387 (36.8%)	619 (34.1%)	
Never	131 (16.4%)	228 (12.6%)	
When you had period pain in the last 3 months how often did this pain get better or stop after you had a bowel movement?			
Always	22 (1.9%)	30 (1.5%)	0.463
Most of the times	82 (7.2%)	161 (8%)	
Often	162 (14.2%)	272 (13.4%)	
Sometimes	439 (38.5%)	740 (36.6%)	
Rarely or never	434 (38.1%)	820 (40.5%)	
When you had period pain in the last 3 months how often did you have more frequent bowel movements?			
Always	13 (1.1%)	18 (0.9%)	0.709
Most of the times	61 (5.4%)	95 (4.7%)	
Often	138 (12.1%)	241 (11.9%)	
Sometimes	376 (33%)	709 (35%)	
Rarely or never	551 (48.4%)	960 (47.5%)	
When you had period pain in the last 3 months how often did you have less frequent bowel movements?			
Always	54 (4.7%)	41 (2%)	**>0.001**
Most of the times	127 (11.2%)	174 (8.6%)	
Often	196 (17.2%)	359 (17.7%)	
Sometimes	354 (31.1%)	645 (31.9%)	
Rarely or never	408 (35.8%)	804 (39.7%)	
When you had period pain in the last 3 months were your stools (bowel movements%) looser?			
Always	60 (5.3%)	67 (3.3%)	**0.001**
Most of the times	174 (15.3%)	237 (11.7%)	
Often	201 (17.3%)	341 (16.9%)	
Sometimes	303 (26.6%)	573 (28.3%)	
Rarely or never	401 (35.2%)	805 (39.8%)	
When you had period pain in the last 3 months were your stools (bowel movements%) harder?			
Always	11 (1%)	31 (1.5%)	0.575
Most of the times	61 (5.4%)	107 (5.3%)	
Often	128 (11.2%)	211 (10.4%)	
Sometimes	324 (28.4%)	551 (27.2%)	
Rarely or never	615 (54%)	1123 (55.5%)	
Pain score as average (mean, SD)	5.79 (2.3)	5.53 (2.41)	0.002
Pain score at worst cases (mean, SD)	6.93 (2.3)	6.68 (2.38)	0.006
Days of bleeding (mean, SD)	5.83 (2.41)	5.66 (1.84)	0.046

*Note*: Statistically significant values are presented in bold.

^a^ Valid percent.

To sum the difference up, females who had received one or more dose of vaccination reported a higher frequency of back pain, nausea, tiredness, pelvic pain with periods, unprescribed analgesics use, and passage of loose stools. They also reported higher scores describing average and worst menstrual pain. Only fully vaccinated females had heavier flow and more days of bleeding.

Vaccinated females with Moderna and Pfizer vaccines had the highest average pain score during menstruation (6.43 and 5.94, respectively) significantly higher than other vaccines. They also had significantly higher number of bleeding days (5.92 for Pfizer and 5.76 for Moderna) compared to other vaccines. a higher percentage of menstrual irregularity was observed in Johnson & Johnson, followed by Sinopharm, Moderna, and AstraZeneca (*p* = 0.022). Similarly, Johnson & Johnson was associated with a higher percentage of heavy bleeding with coagulations, followed by Pfizer, Sinopharm, AstraZeneca, and Moderna (*p* = 0.003). Rectal bleeding was more common in participants vaccinated with Moderna, Pfizer, and Johnson & Johnson (*p* = 0.026). The rest of the menstruation experience did not show significant differences among different vaccine groups. Full comparison between vaccines is written in Table 4.

**TABLE 4:** The comparison of different types of vaccines effect on menstrual experience

	AstraZeneca *N* (%)^a^	J&J *N* (%)^a^	Moderna *N* (%)^a^	Pfizer *N* (%)^a^	Sinopharm *N* (%)^a^	Sputnik *N* (%)^a^	*p* value
Avoid sports due to pelvic pain							0.116
Yes	60 (14.1%)	18 (20.9%)	5 (17.9%)	116 (20.9%)	89 (18.3%)	57 (16.3%)	
No	365 (85.9)	68 (79.1%)	23 (82.1%)	438 (79.1%)	398 (81.7%)	293 (83.7%)	
Avoid period due to period starts							0.555
Yes	193 (41.3%)	40 (43.5%)	16 (47.1%)	248 (42.3%)	192 (37.4%)	157 (40.7%)	
No	274 (58.7%)	52 (56.5%)	18 (52.9%)	338 (57.7%)	322 (62.6%)	229 (59.3%)	
Period regular							0.022
Yes	543 (76.6%)	97 (72.4%)	41 (80.4%)	625 (78.7%)	514 (71.2%)	404 (79.4%)	
No	80 (11.3%)	22 (16.4%)	7 (13.7%)	85 (10.7%)	100 (13.9%)	55 (10.8%)	
Not sure	86 (12.1%)	15 (11.2%)	3 (5.9%)	84 (10.6%)	108 (15%)	50 (9.8%)	
Heaviness of bleed							0.003
Light	52 (7.3%)	6 (4.5%)	7 (13.7%)	70 (8.8%)	65 (9%)	45 (8.8%)	
Moderate	478 (67.4%)	92 (68.7%)	32 (62.7%)	520 (65.5%)	459 (63.6%)	370 (72.7%)	
Heavy with coagulations	155 (21.9%)	33 (24.6%)	11 (21.6%)	189 (23.8%)	162 (22.4%)	85 (16.7%)	
I do not remember	24 (3.4%)	3 (2.2%)	1 (2%)	15 (1.9%)	36 (5%)	9 (1.8%)	
How many days are there between the start of one period and the start of the next on average?							0.076
Less than 21 days	40 (5.6%)	7 (5.2%)	5 (9.8%)	45 (5.7%)	32 (4.4%)	22 (4.3%)	
22–24 days	135 (19%)	31 (23.1%)	12 (23.5%)	180 (22.7%)	129 (17.9%)	86 (16.9%)	
25–28 days	290 (40.9%)	53 (39.6%)	17 (33.3%)	308 (38.8%)	287 (39.8%)	216 (42.4%)	
29–32 days	28 (3.9%)	25 (18.7%)	11 (21.6%)	154 (19.4%)	153 (21.2%)	124 (24.4%)	
33–35 days	28 (3.9%)	4 (3%)	2 (3.9%)	40 (5%)	42 (5.8%)	30 (5.9%)	
More than 36 days	10 (1.4%)	5 (3.7%)	1 (2%)	22 (2.8%)	13 (1.8%)	5 (1%)	
Cannot determine due to irregularity	56 (7.9%)	9 (6.7%)	3 (5.9%)	45 (5.7%)	66 (9.1%)	26 (5.1%)	
Do you have pelvic pain							0.079
Yes	615 (86.7%)	109 (81.3%)	45 (88.2%)	654 (82.4%)	602 (83.4%)	442 (86.8%)	
No	94 (13.3%)	25 (18.7%)	6 (11.8%)	140 (17.6%)	120 (16.6%)	67 (13.2%)	
Do you have anal pain							0.121
Yes	148 (20.9%)	21 (15.7%)	9 (17.6%)	189 (23.8%)	151 (20.9%)	93 (18.3%)	
No	561 (79.1%)	113 (84.3%)	42 (82.4%)	605 (76.2%)	571 (79.1%)	416 (81.7%)	
Do you have anal bleeding							0.026
Yes	51 (7.2%)	13 (9.7%)	7 (13.7%)	89 (11.2%)	56 (7.8%)	36 (7.1%)	
No	658 (92.8%)	121 (90.3%)	44 (86.3%)	705 (88.8%)	666 (92.2%)	473 (92.9%)	
Do you have pain at urination							0.142
Yes	98 (13.8%)	18 (13.4%)	10 (19.6%)	123 (15.5%)	119 (16.5%)	58 (11.4%)	
No	611 (86.2%)	116 (86.6%)	41 (80.4%)	671 (84.5%)	603 (83.5%)	451 (88.6%)	
Do you have bleeding at urination							0.068
Yes	181 (25.5%)	39 (29.1%)	20 (39.2%)	236 (29.7%)	218 (30.2%)	128 (25.1%)	
No	528 (74.5%)	95 (70.9%)	31 (60.8%)	558 (70.3%)	504 (69.8%)	381 (74.9%)	
Do you have back pain							0.539
Yes	575 (81.1%)	111 (82.8%)	45 (88.2%)	665 (83.8%)	595 (82.4%)	430 (84.5%)	
No	134 (18.9%)	23 (17.2%)	6 (11.8%)	129 (16.2%)	127 (17.6%)	79 (15.5%)	
Do you have thigh pain							0.366
Yes	443 (62.5%)	75 (56%)	34 (66.7%)	513 (64.6%)	473 (65.5%)	326 (64%)	
No	266 (37.5%)	59 (44%)	17 (33.3%)	281 (35.4%)	249 (34.5%)	183 (36%)	
Do you have nausea							0.145
Yes	304 (42.9%)	61 (45.5%)	26 (51%)	366 (46.1%)	298 (41.3%)	201 (39.5%)	
No	405 (57.1%)	73 (54.5%)	25 (49%)	428 (53.9%)	424 (58.7%)	308 (60.5%)	
Do you have tiredness							0.211
Yes	642 (90.6%)	125 (93.3%)	48 (04.1%)	713 (89.8%)	632 (87.5%)	457 (89.8%)	
No	67 (9.4%)	9 (6.7%)	3 (5.9%)	81 (10.2%)	90 (12.5%)	52 (10.2%)	
In the last 3 months, have you had pelvic pain with your periods?							0.289
Yes	622 (87.7%)	113 (84.3%)	43 (84.3%)	665 (83.8%)	613 (84.9%)	443 (87%)	
No	87 (12.3%)	21 (15.7%)	8 (15.7%)	129 (16.2)	109 (15.1%)	66 (13%)	
How often have you had pelvic pain with your periods in the last 3 months?							0.276
Sometimes	153 (23.9%)	19 (16.8%)	9 (19.6%)	153 (22.2%)	127 (19.9%)	122 (26.7%)	
Usually	142 (22.2%)	21 (18.6%)	10 (21.7%)	151 (21.9%)	146 (22.9%)	92 (20.1%)	
Always	345 (53.9%)	73 (64.6%)	27 (58.7%)	385 (55.9%)	364 (57.1%)	243 (53.2%)	
In the last 3 months, have you taken pain‐killers for the pain, bought over the counter without prescription?							0.367
Yes	434 (65.4%)	72 (61%)	26 (57.8%)	460 (63.8%)	406 (61.9%)	279 (59.4%)	
No	230 (34.6%)	46 (39%)	19 (42.2%)	261 (36.2%)	250 (38.1%)	191 (40.6%)	
In the last 3 months, have you taken pain‐killers for the pain that are prescribed for you by a doctor?							0.519
Yes	70 (10.6%)	15 (13%)	8 (17.8%)	92 (12.8%)	72 (11%)	50 (10.7%)	
No	592 (89.4%)	100 (87%)	37 (82.2%)	625 (87.2%)	581 (89%)	416 (89.3)	
In the last 3 months, has your period pain prevented you from going to work or carrying out your daily activities (even if taking pain‐killers)?							0.402
Occasionally (with 1 in 3 of my periods)	234 (35.1%)	39 (33.1%)	17 (36.2%)	240 (33.1%)	227 (34.7%)	139 (29.8%)	
Often (with 2 in 3 of my periods)	80 (12%)	16 (13.6%)	9 (19.1%)	93 (12.8%)	93 (14.2%)	52 (11.1%)	
Always (with every period)	68 (10.2%)	17 (14.4%)	5 (10.6%)	81 (11.2%)	71 (10.9%)	46 (9.9%)	
Never	284 (42.6%)	46 (39%)	16 (34%)	310 (42.8%)	263 (40.2%)	230 (49.3%)	
In the last 3 months, have you had to lie down for any part of the day or longer because of your period pain?							0.575
Occasionally (with 1 in 3 of my periods)	213 (31.9%)	30 (25.2%)	12 (26.1%)	217 (30%)	195 (29.5%)	146 (31.1%)	
Often (with 2 in 3 of my periods)	148 (22.2%)	28 (23.5%)	13 (28.3%)	156 (21.6%)	150 (22.7%)	99 (21.2%)	
Always (with every period)	239 (35.8%)	48 (40.3%)	18 (39.1%)	264 (36.5%)	236 (35.7%)	151 (32.3%)	
Never	67 (10%)	13 (10.9%)	3 (6.5%)	86 (11.9%)	80 (12.1%)	71 (15.2%)	
When you had period pain in the last 3 months how often did this pain get better or stop after you had a bowel movement?							0.445
Always	15 (2.1%)	2 (1.5%)	0 (0%)	10 (1.3%)	11 (1.5%)	11 (2.2%)	
Most of the times	50 (7.1%)	7 (5.2%)	4 (7.8%)	67 (8.4%)	29 (5.7%)	29 (5.7%)	
Often	104 (14.7%)	24 (17.9%)	12 (23.5%)	112 (14.1%)	79 (15.5%)	79 (15.5%)	
Sometimes	277 (39.1%)	51 (38.1%)	13 (25.5%)	301 (37.9%)	187 (36.7%)	187 (36.7%)	
Rarely or never	263 (37.1%)	50 (37.3%)	22 (43.1%)	304 (38.3%)	298 (41.3%)	203 (39.9%)	
When you had period pain in the last 3 months how often did you have more frequent bowel movements?							0.711
Always	8 (1.1%)	1 (0.7%)	2 (3.9%)	5 (0.6%)	10 (1.4%)	6 (1.2%)	
Most of the times	35 (4.9%)	7 (5.2%)	1 (2%)	46 (5.8%)	43 (6%)	21 (4.1%)	
Often	72 (10.2%)	14 (10.4%)	5 (9.8%)	92 (11.6%)	87 (12%)	63 (12.4%)	
Sometimes	241 (34%)	42 (31.3%)	21 (41.2%)	280 (35.3%)	256 (35.5%)	168 (33%)	
Rarely or never	353 (49.8%)	70 (52.2%)	22 (43.1%)	371 (46.7%)	326 (45.2%)	251 (49.3%)	
When you had period pain in the last 3 months how often did you have less frequent bowel movements?							0.465
Always	29 (4.1%)	1 (0.7%)	1 (2%)	30 (3.8%)	25 (3.5%)	15 (2.9%)	
Most of the times	84 (11.8%)	16 (11.9%)	9 (17.6%)	77 (9.7%)	67 (9.3%)	52 (10.2%)	
Often	136 (19.2%)	25 (18.7%)	10 (19.6%)	131 (16.5%)	113 (15.7%)	74 (14.5%)	
Sometimes	215 (30.3%)	42 (31.3%)	17 (33.3%)	261 (32.9%)	244 (33.8%)	176 (34.6%)	
Rarely or never	245 (34.6%)	50 (37.3%)	14 (27.5%)	295 (37.2%)	273 (37.8%)	192 (37.7%)	
When you had period pain in the last 3 months were your stools (bowel movements) looser?							0.305
Always	28 (3.9%)	4 (3%)	2 (3.9%)	45 (5.7%)	34 (4.7%)	21 (4.1%)	
Most of the times	108 (15.2%)	22 (16.4%)	7 (13.7%)	112 (14.1%)	82 (11.4%)	58 (11.4%)	
Often	141 (19.9%)	32 (23.9%)	13 (25.5%)	141 (17.8%)	120 (16.6%)	94 (18.5%)	
Sometimes	186 (26.2%)	28 (20.9%)	12 (23.5%)	204 (25.7%)	209 (28.9%)	133 (26.1%)	
Rarely or never	246 (34.7%)	48 (35.8%)	17 (33.3%)	292 (36.8%)	277 (38.4%)	203 (39.9%)	
When you had period pain in the last 3 months were your stools (bowel movements) harder?							**0.001**
Always	6 (0.8%)	0 (0%)	0 (0%)	10 (1.3%)	11 (1.5%)	6 (1.2%)	
Most of the times	39 (5.5%)	13 (9.7%)	3 (5.9%)	42 (5.3%)	38 (5.3%)	24 (4.7%)	
Often	68 (9.6%)	7 (5.2%)	8 (15.7%)	88 (11.1%)	90 (12.5%)	46 (9%)	
Sometimes	202 (28.5%)	32 (23.9%)	10 (19.6%)	209 (26.3%)	226 (31.3%)	106 (20.8%)	
Rarely or never	394 (55.6%)	82 (61.2%)	30 (58.8%)	445 (56%)	357 (49.4%)	327 (64.2%)	
Pain score as average	5.72 (2.2)	5.73 (2.59)	6.43 (2.22)	5.94 (2.4)	5.87 (2.25)	5.5 (2.42)	**0.007**
Pain score at worst cases	6.97 (2.26)	6.88 (2.48)	7.52 (1.9)	7.06 (2.37)	6.97 (2.31)	6.75 (2.38)	0.106
Days of bleeding	5.70 (2.65)	5.47 (2.1)	5.76 (1.58)	5.92 (1.75)	5.53 (2.03)	5.62 (1.5)	**<0.001**

*Note*: Statistically significant values are presented in bold.

^a^ Valid percent.

The group of females who were vaccinated and have a history of COVID‐19 had a significantly higher heaviness of bleeding, a higher prevalence of pelvic pain, anal pain, back pain, thigh pain, general weakness, menstrual pain prevalence, use of analgesics, and higher pain scores. A full table to compare the four groups including vaccinated and had COVID‐19, vaccinated with no history of COVID‐19, not vaccinated with a history of COVID‐19, and not vaccinated with no history of COVID‐19 is found as Supporting Information S2. In addition, a post hoc analysis was performed to assess difference between mentioned groups, results showed that group one had the highest pain score on average, pain score in worst cases, and highest days of bleeding; this indicated an association between pain scores and both vaccination and COVID‐19 infection. Full table for post hoc analysis is available in Table 5.

**TABLE 5:** Post hoc ANOVA test to compare four groups in this study

	Vaccinated and had COVID19 mean (SD)	Vaccinated with no history of COVID19 mean (SD)	Not vaccinated with history of COVID19 mean (SD)	Not vaccinated with no history of COVID19 mean (SD)
Pain score as average	5.86 (2.29)^a^	5.76 (2.35)^a,b^	5.62 (2.41)^b,c^	5.45 (2.4)^c^
Pain score at worst cases	7.08 (2.29)^a^	6.9 (2.35)^a^	6.86 (2.29)^a^	6.55 (2.43)^b^
Days of bleeding	5.84 (2.6)^a^	5.61 (1.7)^b^	5.68 (1.64)^a,b^	5.64 (1.97)^b^

*Note*: Means that carry the same letter in the same raw are not statistically significant.

In all the three regression models applied on the data, receiving the vaccine was a significant predictor for higher frequency of all of pelvic pain, back pain, nausea, general weakness, menstrual pain, unprescribed analgesics use, more frequent bowel movement, and more loose stool status, after adjustment for demographics. Full analysis can be found in Supporting Information S3.

## 4 DISCUSSION

This study is one of the first studies to assess the effect of the COVID‐19 vaccine on menstrual experience among females in the middle‐east region and worldwide. Same outcomes were assessed for both females who received the the COVID‐19 vaccine and females who did not receive any vaccine doses to avoid any subjective reviews by considering females who did not receive the COVID‐19 vaccine as a control group. This study revealed that receipt of the COVID‐19 vaccine was significantly associated with increased back pain, nausea, tiredness, pelvic pain with periods, administration of over the counter analgesics, bowel movement, looseness of the stool, and pain score on average. An increase in the heaviness of bleeding was reported among females who were fully vaccinated.

It was critical to perform menstrual health research in the context of the COVID‐19 pandemic considering the strong relationship between regular normal cycles and the general health of females. Irregular menstrual cycles has been linked to an increased risk of breast and ovarian cancer, early menopause, infertility, chronic renal failure, diabetes mellitus, and cardiovascular disease,^12^, ^13^, ^14^, ^15^ furthermore, having menstrual health issues may have a significant impact on quality of life.^16^ In addition, women's menstrual health might be adversely affected by situations of emotional or physical stress, which can result in a condition known as functional hypothalamic amenorrhea (FHA), in which there is no underlying biological cause of anovulation.^17^, ^18^ In addition to missing periods, psychological distress has been linked to aggravation of menstrual and psychosexual health complications. Depression, emotional instability, and high‐stress levels have been linked to dysmenorrhea.^19^, ^20^ Menorrhagia and premenstrual symptoms (PMS) have also been linked to significant levels of psychological distress.^21^, ^22^

Based on the findings of this study, a direct comparison between participants who have been vaccinated against COVID‐19 and those who have not been vaccinated showed that females who were vaccinated had a higher frequency of back pain, nausea, tiredness, pelvic pain with periods, unprescribed analgesics use, and passage of loose stools. They also had higher scores reported describing average and worst menstrual pain. By September 2021, 30 000 reports of changes in menstrual cycle were reported to the Medicines and Healthcare Products Regulatory Agency (MHRA) although no reported side effect that the vaccine can affect the menstrual cycle were found.^8^ A study conducted in the MENA region among Palestine, Iraq, Lebanon, Al‐Bahrain, Tunisia, Kuwait, Qatar, Turkey, Jordan, UAE, KSA, Egypt, Oman, Morocco, Sudan, and Syria on the effect of COVID‐19 vaccine in the menstrual experience indicated that 66.3% of females experienced menstrual abnormalities after getting vaccinated with COVID‐19 vaccine.^23^ Another study conducted by Laganà et. al, indicated that 50%–60% of participants have reported menstrual cycle irregularities after receiving the first dose of COVID‐19 vaccine.^24^

In a prospective study that aimed to investigate the impact of the COVID‐19 pandemic on the reproductive system of women, authors reported an overall menstrual change of 46%, and that 53% experienced worse premenstrual symptoms, 49% experienced painful periods, 47% reported heaviness of bleeding, 45% had decreased libido, 29% had increased period length, 28% experienced reduced period length, and 9% reported new missed periods.^25^ A cross‐sectional study of 200 women in Jordan found a substantial decrease in menstruation disorders during the COVID‐19 lockdown (*p* = 0.016), pre‐curfew, curfew, and post‐curfew, and access to healthcare facilities for menstruation difficulties did not change statistically, however phone consultations increased dramatically during the curfew.^26^ Since Jordan's curfew only lasted a few days, some researchers hypothesized that the population's stress levels were not high enough to cause additional menstruation irregularities. On the other hand, an observational study in Turkey revealed an increase in menstrual abnormalities during the lockdown vs. pre‐lockdown (*p* = 0.008), although these results should be interpreted with caution, as the study included only 58 participants.^27^ According to previously published studies, premenstrual symptoms are more common in women with a high degree of psychosocial stress.^18^ A large percentage of women with heavy and painful periods is expected, given both have been linked to stress, psychological distress, and depressed mood.^19^, ^20^, ^21^, ^28^

When it comes to COVID‐19 infection, in China, a single‐center retrospective study compared menstrual patterns among mildly and severely ill women with COVID‐19.^29^ Menstrual bleeding was observed to be reduced in 20% of women who had confirmed COVID‐19 infection, in addition, compared to a control group, COVID‐19 patients had an increased menstrual volume and menstrual cycle abnormalities.^29^ As previously reported, anovulation occurs in a variety of acute disorders to ensure that vital organs operate properly.^30^

By comparing the six vaccine types, a higher percentage of menstrual irregularity was observed in Johnson & Johnson, followed by Sinopharm, Moderna, and AstraZeneca (*p* = 0.022). Similarly, Johnson & Johnson was associated with a higher percentage of heavy bleeding with coagulations, followed by Pfizer, Sinopharm, AstraZeneca, and Moderna (*p* = 0.003). Rectal bleeding was more common in participants vaccinated with Moderna, Pfizer, and Johnson & Johnson (*p* = 0.026). Moreover, binary logistic regression showed that COVID‐19 vaccination was associated with an increased risk of pelvic pain, back pain, thigh pain, nausea, general weakness, menstrual pain, receiving analgesics for menstrual pain, experiencing bowel movement more than usual, and experiencing stool more liquid than usual. In a large observational study of 3959 participants (61% vaccinated and 39% non‐vaccinated), authors aimed to determine the impact of COVID‐19 vaccination on the menstrual cycle in those receiving vaccination as compared with an unvaccinated cohort. They showed that the Pfizer‐BioNTech vaccine was used by the majority of the vaccinated group (55%), followed by Moderna (35%) and Johnson & Johnson (7%). Their findings showed that the COVID‐19 vaccine was linked to a minimal change in cycle length (less than 1 day) compared with pre‐vaccine cycles. There was no substantial change in the three baseline cycles for unvaccinated individuals. The difference in cycle duration between the vaccinated and unvaccinated populations was less than 1 day in adjusted models for both dosages (first and second doses). Finally, they concluded that COVID‐19 vaccination was associated with a small change in cycle length but not menses length.^9^ mRNA vaccinations might cause a strong immunological response or stressor, which can temporarily disrupt the hypothalamic–pituitary‐ovarian axis.^31^, ^32^, ^33^ Given the dosage schedule for the mRNA COVID‐19 vaccines in certain countries (21 days for Pfizer and 28 days for Moderna), a person getting two doses in a single cycle would have received the first dose during the early follicular phase. During the follicular phase, factors that contribute to the recruitment and maturation of the dominant follicle are known to impact cycle duration variability.^34^, ^35^

Our study has a number of limitations; first of all, a potential limitation of the study may arise from the cross‐sectional study design that prevents any conclusions regarding the causal relationships between the COVID19 vaccine and psychological menstrual disturbances, in addition to that, the online nature of the data collection and the convenience sampling of the study participants stand as a limitation regarding the generalizability of the results, and finally, the pool of the concluded countries lacks the representation of some countries in the MENA region as the gulf countries.

In conclusion, the current evidence suggests that the COVID‐19 vaccine significantly affects the menstrual cycle in terms of increasing pain score, aggravating the menstrual pain including back pain, pelvic pain, thigh pain, increasing the heaviness of bleeding, and changing the bowel movement, which collectively affects the quality of life of infected women, however, further data are required to confirm this finding. Nevertheless, COVID‐19 vaccination can reduce the risk of infection and severe disease and numerous studies have confirmed that the benefits of vaccination outweigh the risks.

## CONFLICTS OF INTEREST

The authors declare no conflict of interest.

## ETHICS STATEMENT

Ethical approval of research (No. 2022‐PHA‐1) was obtained from the Institutional Review Board (IRB) of the Applied Science Private University (ASU), Jordan. Informed consent was obtained from all participants. All methods were performed in accordance with the relevant guidelines and regulations.

## AUTHOR CONTRIBUTIONS

**Sajeda Ghassan Matar:** Conceptualization; data curation; formal analysis; investigation; methodology; project administration; resources; software; supervision; validation; visualization; writing‐original draft; writing‐review and editing. **Anas Zakarya Nourelden:** Formal analysis. **Ahmed Assar:** Conceptualization; investigation; methodology. **Eshak I. Bahbah:** Conceptualization. **Areej M. Alfryjat:** Conceptualization. **Elfatih A. Hasabo:** Conceptualization; investigation; project administration; writing‐original draft; writing‐review and editing. **Suzan A. Matar:** Conceptualization; writing‐review and editing. **Shatha Nizar Bishtawi:** Conceptualization; writing‐original draft. **Mays Alhoubani:** Formal analysis. **Ahmad Bassam Yahia:** Conceptualization. **Khaled Mohamed Ragab:** Conceptualization. **Lina Mohammad Salameh:** Conceptualization. **Lana Saif Eddin Salameh:** Conceptualization. **Mohamed Sayed Zaazouee:** Conceptualization. **Mohammed Al‐kafarna:** Data curation. **Alaa Ahmed Elshanbary:** Data curation. **Hossam Waleed Almadhoon:** Data curation. **Shahed Toulaq Bakdash:** Data curation. **Ola Awad Babiker Adam:** Data curation. **Abdelkader Nabeel Malih:** Data curation. **Shimaa Abo elfotoh Habash:** Data curation. **Rakia Mohamed Taha Basiouny:** Data curation. **Afaf Ahmad:** Data curation. **Raghda Mohammed Ahmed Hamid:** Data curation. **Balsam Younan Habib:** Data curation. **Dalia Nasr Elokl:** Data curation. **Hiba Hatim Abdalraheem:** Data curation. **Esraa Adel Atia:** Data curation. **Nazik Ibrahim Ahmed Yousif:** Data curation. **Fida Hussien Al‐Ali:** Data curation. **Israa mohammed Alshaer:** Data curation. **Fatima Elsidieg Abdulali:** Data curation. **Hadil Abu Ayesh:** Data curation. **Anwar Yousef Jabari:** Data curation. **Raneem Ahmed Egzait:** Data curation. **Nameer Amer Abu Munshar:** Data curation. **Aseel Ahmad Alkhraibat:** Data curation. **Aisha Hasan ibreerah:** Data curation. **Iman A. Basheti:** Conceptualization; data curation; project administration; supervision; writing‐review and editing.

### PEER REVIEW

The peer review history for this article is available at https://publons.com/publon/10.1111/irv.13088.

## Supporting information


**Supporting Information S1.** The questionnaire

**Supporting Information S2.** Comparison between four groups of participants: Click here for additional data file.

**Supporting Information S3.** Effect of demographic characteristics on menstrual experience (a binary logistic regression)

## Data Availability

The data that support the findings of this study are available on request from the corresponding author. The data are not publicly available due to privacy or ethical restrictions.
