# Supplementary material for: Effect of COVID‐19 vaccine on menstrual experience among females in six Arab countries: A cross sectional study
Source: Influenza Other Respir Viruses. 2022 Dec 28;17(1):e13088. doi: 10.1111/irv.13088 (PMC9835440; doi:10.1111/irv.13088)
Supplement: Supplementary file 1 — Supporting Information S1. The questionnaire [file IRV-17-0-s003.docx]

**Supporting Information S1: the questionnaire**

**Title: Effect of COVID19 vaccine on menstruation in the middle east- a cross sectional study**

**This survey is voluntary. You may decline to answer any of the questions. By checking the box below, you are acknowledging that you are voluntarily participating in this survey, have read, and understood the Information Sheet, and consent**

I consent

I do not consent

**Are you: *Pregnant or *breast-feeding and/or *administer oral contraceptives, and/or have *intra-uterine device, and/or have *PCOS or *fibroids, and/or *receive hormone replacement therapy, and/or *menopause, and/or *bleed between two cycles?**

Yes

No

**COVID19 vaccination and menstruation experience**

**Age**

**Country**

Egypt

Jordan

Syria

Palestine

Libya

Alegria

Sudan

**Residency**

Rural

Urban

Education

None

Elementary

Preparatory (Middle school)

Secondary (High school)

Bachelor degree

Advanced degree (MSc, PhD)

**Are you a healthcare professional (Physician, Nurse, Pharmacist, Dentist, etc.) or a medical student?**

Yes

No

**Employment status**

Full time

Part time

Unemployed

**Weight in Kgs**

**Height in cm**

**Do you smoke?**

Yes

No

Sometimes

**Number of cigarettes per day**

**have you been experiencing stressful conditions in the last three months?**

Yes

No

Maybe

**Have you ever done any vigorous leisure exercise or sports (i.e., exercise that made you breathe faster, such as jogging, swimming, cycling or aerobic exercise)?**

Yes

No

**If the answer was yes, in the last 3 months, how often did you do vigorous exercise or sports?**

Never

Occasionally (2-3 times a month)

Regularly (about once a week)

Often (a few times a week)

Every day

Can’t remember

**In the last 3 months, did you avoid vigorous exercise at certain times, because of……**

|  | Yes | No |
| --- | --- | --- |
| Pelvic pain? |  |  |
| Having a period? |  |  |

**At what age did you have your first period?**

**Are your periods regular? (Predictable within one week)**

Yes

No

Not sure

**How many days of bleeding do you usually have each period?**

**How heavy is your menstrual flow usually?**

Light

moderate

Heavy (clots)

Can't remember

**How many days are there between the start of one period and the start of the next on average?**

Less than 21 days

22-24 days

25-28 days

29-32 days

33-35 days

More than 36 days

Too irregular to say

**Do you have any of the following symptoms when you have a period?**

|  | Yes | No |
| --- | --- | --- |
| Pelvic pain (pain in the lower party of your belly) |  |  |
| Pain on opening your bowels |  |  |
| Bleeding from your back passage when opening your bowels |  |  |
| Pain on passing urine |  |  |
| Passing blood in your urine |  |  |
| Lower back pain |  |  |
| Pain in upper leg or thighs |  |  |
| Nausea |  |  |
| Tiredness |  |  |

**In the last 3 months, have you had pelvic pain with your periods?** By ‘pelvic pain’ we mean any type of pain in the lower part of your belly (the area from your navel down)

Yes

No

**If the previous answer was yes, please answer the following questions regarding pelvic pain.**

**If yes, how often have you had pelvic pain with your periods in the last 3 months?**

Occasionally (with 1 in 3 of my periods)

Often (with 2 in 3 of my periods)

Always (with every period)

**In the last 3 months, have you taken pain-killers for the pelvic pain that are prescribed for you by a doctor?**

Yes

No

**In the last 3 months, have you taken pain-killers for the pelvic pain, bought over the counter without prescription?**

Yes

No

**In the last 3 months, has your period pelvic pain prevented you from going to work or carrying out your daily activities (even if taking pain-killers)?**

Never

Occasionally (with 1 in 3 of my periods)

Often (with 2 in 3 of my periods)

Always (with every period)

**In the last 3 months, have you had to lie down for any part of the day or longer because of your period pelvic pain?**

Never

Occasionally (with 1 in 3 of my periods)

Often (with 2 in 3 of my periods)

Always (with every period)

**Please circle on the following scale, going from no pain (0) to worst possible pain (10), the number that indicates how severe your period pain has been ON AVERAGE in the last 3 months:**

| 0 | 1 | 2 | 3 | 4 | 5 | 6 | 7 | 8 | 9 | 10 |
| --- | --- | --- | --- | --- | --- | --- | --- | --- | --- | --- |
| No pain |  |  |  |  |  |  |  |  |  | Worst possible pain |

**Please circle on the following scale, going from no pain (0) to worst possible pain (10), the number that indicates how severe your period pain has been AT ITS WORST in the last 3 months:**

| 0 | 1 | 2 | 3 | 4 | 5 | 6 | 7 | 8 | 9 | 10 |
| --- | --- | --- | --- | --- | --- | --- | --- | --- | --- | --- |
| No pain |  |  |  |  |  |  |  |  |  | Worst pain ever |

**The following questions are about your bowel movements/stool when you had period pain IN THE LAST 3 MONTHS**

|  | Never/Rarely | Sometimes | Often | Most of the time | Always |
| --- | --- | --- | --- | --- | --- |
| how often did this pain get better or stop after you had a bowel? |  |  |  |  |  |
| how often did you have more frequent bowel movements? |  |  |  |  |  |
| how often did you have less frequent bowel movements? |  |  |  |  |  |
| were your stools (bowel movements) looser? |  |  |  |  |  |
| were your stools (bowel movements) harder? |  |  |  |  |  |

**Have you received COVID-19 vaccine?**

*

Yes

No

**I received COVID-19 vaccine**

**Vaccine type**

Pfizer

AstraZeneca

Sinopharm

Sputnik V

Moderna

Johnson and Johnson

**Have you received**

Only first dose

Both doses

Both doses and a third dose

Johnson and Johnson vaccine

**Since when you have received your first dose?** (the answer should be in numbers in months: 1 months, 1.5 months, 2 months.. etc.)

**since when have you received your second dose (IF received)?** (the answer should be in numbers in months: 1 months, 1.5 months, 2 months.. etc.)

**After receiving COVID-19 vaccine, have you noticed any changes in your menstrual experience in the following aspects?**

|  | Yes | No | It did not change |
| --- | --- | --- | --- |
| Duration of my period is becoming longer |  |  |  |
| Heaviness of my period increased |  |  |  |
| Pelvic pain has increased |  |  |  |

**Have you ever had COVID-19 infection before?**

Yes

No

**COVID-19 demographics**

**I only had mild symptoms (Cough, fatigue, muscle pain, fever, loss of smell, etc.)**

Yes

I had no symptoms

I had those symptoms in addition to other more severe symptoms

**Oxygen saturation was below 90% at sometimes**

Yes

No

Not sure

**I had pneumonia**

Yes

No

I am not sure

**I required oxygen therapy**

Yes

No

I am not sure

**I required mechanical ventilation**

Yes

No

I am not sure

**During my COVID-19 infection I was**

Isolated at home

Hospitalized in the floor

Admitted to ICU

**After recovery from COVID-19, have you noticed any changes in your menstrual experience in the following aspects?**

|  | Yes | No | It did not change |
| --- | --- | --- | --- |
| Duration of my period is becoming longer |  |  |  |
| Heaviness of my period increased |  |  |  |
| Pelvic pain has increased |  |  |  |

**Thank you for participating**
