# Supplementary material for: Effect of COVID‐19 vaccine on menstrual experience among females in six Arab countries: A cross sectional study
Source: Influenza Other Respir Viruses. 2022 Dec 28;17(1):e13088. doi: 10.1111/irv.13088 (PMC9835440; doi:10.1111/irv.13088)
Supplement: Supplementary file 2 — Supporting Information S2. Comparison between four groups of participants: [file IRV-17-0-s002.docx]

**Supporting Information S2: Comparison between four groups of participants:**

|  | **COVID19 and Vaccinated**  **N (%)** | **Vaccinated and No COVID19** | **Not Vaccinated had COVID19** | **No Vaccine No COVID19** | **P-value** |
| --- | --- | --- | --- | --- | --- |
| **Is your period Regular** | | | | | |
| **Yes** | 766 (77.8%) | 1458 (75.3%) | 618 (72.4%) | 826 (70.7%) | **0.005** |
| **No** | 115 (11.7%) | 234 (12.1%) | 122 (14.3%) | 180 (15.4%) |  |
| **Not Sure** | 103 (10.5%) | 243 (12.6%) | 114 (13.3%) | 163 (13.9%) |  |
| **Heaviness of menstrual bleeding** | | | | | |
| - Mild | 97 (8.0%) | 166 (8.6%) | 85 (10.0% | 78 (6.7%) | **<0.001** |
| - Moderate | 644 (65.4%) | 1307 (67.5%) | 548 (64.2%) | 778 (66.6%) |  |
| - Heavy | 244 (24.8%) | 391 (20.2%) | 196 (23.0%) | 250 (21.4%) |  |
| - Can’t remember | 17 (1.7%) | 71 (3.7%) | 25 (2.9%) | 63 (5.4%) |  |
| **Pelvic Pain** | | | | | |
| - Yes | 844 (85.8%) | 1623 (83.9%) | 715 (83.7%) | 935 (80.0%) | **0.003** |
| - No | 140 (14.2%) | 312 (16.1%) | 139 (16.3%) | 234 (20.0%) |  |
| **Anal Pain** | | | | | |
| - Yes | 232 (23.6%) | 397 (19.6%) | 191 (22.4%) | 191 (16.3%) | **<0.001** |
| - No | 752 (76.4%) | 1556 (80.4%) | 663 (77.6%) | 978 (83.7%) |  |
| **Pain at urination** | | | | | |
| - Yes | 153 (15.5%) | 273 (14.1%) | 132 (15.5%) | 141 (12.1%) | 0.072 |
| - No | 831 (84.5%) | 1662 (85.9%) | 722 (84.5%) | 1028 (87.9%) |  |
| **Back pain** | | | | | |
| - Yes | 856 (87.0%) | 1565 (80.9%) | 687 (80.4%) | 889 (76.0%) | **<0.001** |
| - No | 128 (13.0%) | 370 (19.1%) | 167 (19.6%) | 280 (24.0%) |  |
| **Thigh Pain (Upper leg pain)** | | | | | |
| - Yes | 679 (69.0%) | 1185 (61.2%) | 537 (62.9%) |  | **<0.001** |
| - No | 305 (31.0%) | 750 (38.8%) | 317 (37.1%) |  |  |
| **Nausea** | | | | | |
| - Yes | 450 (45.7%) | 806 (41.7%) | 364 (42.6%) | 446 (38.2%) | **0.005** |
| - No | 534 (54.3%) | 1129 (58.3%) | 490 (57.4%) | 723 (61.8%) |  |
| **General weakness** | | | | | |
| - Yes | 904 (91.9%) | 1713 (88.5%) | 764 (89.5%) | 998 (85.4%) | **<0.001** |
| - No | 80 (8.1%) | 222 (11.5%) | 90 (10.5%) | 171 (14.6%) |  |
| **Pelvic pain in the last 3 months** | | | | | |
| - Yes | 847 (86.1%) | 1652 (85.4%) | 715 (83.7%) | 942 (80.6%) | **<0.001** |
| - No | 137 (13.9%) | 283 (14.6%) | 139 (16.3%) | 227 (19.4%) |  |
| **Menstrual pain prevalence** | | | | | |
| - Sometimes | 185 (21.2%) | 398 (23.3%) | 169 (22.8%) | 308 (30.9%) | **<0.001** |
| - Usually | 192 (22.0%) | 370 (21.6%) | 189 (25.5%) | 241 (24.2%) |  |
| - Always | 494 (56.7%) | 943 (55.1%) | 383 (51.7%) | 448 (44.9%) |  |
| **Analgesics for menstrual pain (without prescription) e.g. Ibuprofen in the last 3 months** | | | | | |
| - Yes | 597 (65.9%) | 1080 (61.1%) | 498 (64.9%) | 538 (51.6%) | **<0.001** |
| - No | 309 (34.1%) | 688 (38.9%) | 269 (35.1%) | 505 (48.4%) |  |
| **Pain preventing from work** | | | | | |
| - Sometimes | 316 (34.8%) | 580 (32.8%) | 255 (33.4%) | 321 (30.6%) | **0.008** |
| - Usually | 121 (13.3%) | 222 (12.5%) | 104 (13.6%) | 117 (11.2%) |  |
| - Always | 105 (11.6%) | 183 (10.3%) | 96 (12.6%) | 96 (9.2%) |  |
| - Never | 365 (40.2%) | 784 (44.3%) | 309 (40.4%) | 514 (49.0%) |  |
| **Bowel movement more than usual** | | | | | |
| - Always | 29 (2.9%) | 72 (3.7%) | 20 (2.3%) | 21 (1.8%) | **0.001** |
| - Most | 108 (11.0%) | 197 (10.2%) | 74 (8.7%) | 100 (8.6%) |  |
| - Usually | 178 (18.1%) | 311 (16.1%) | 164 (19.2%) | 195 (16.7%) |  |
| - Sometimes | 339 (34.5%) | 616 (31.8%) | 285 (33.4%) | 360 (30.8%) |  |
| - Rarely or never | 330 (33.5%) | 739 (38.2%) | 311 (36.4%) | 493 (42.2%) |  |
| **Stool more liquid than usual** | | | | | |
| - Always | 43 (4.4%) | 91 (4.7%) | 34 (4.0%) | 33 (2.8%) | **0.001** |
| - Most | 146 (14.8%) | 243 (12.6%) | 110 (12.9%) | 127 (10.9%) |  |
| - Usually | 187 (19.0%) | 354 (18.3%) | 149 (17.4%) | 192 (16.4%) |  |
| - Sometimes | 271 (27.5%) | 501 (25.9%) | 258 (30.2%) | 315 (26.9%) |  |
| - Rarely or never | 337 (34.2%) | 746 (38.6%) | 303 (35.5%) | 502 (42.9%) |  |
|  | **Vaccinated and had COVID19**  Mean (SD) | **Vaccinated with no history of COVID19**  Mean (SD) | **Not vaccinated with history of COVID19** Mean (SD) | **Not vaccinated with no history of COVID19**  Mean (SD) | **P-value** |
| **Pain score as average** | 5.86 (2.29) | 5.76 (2.35) | 5.62 (2.41) | 5.45 (2.4) | **<0.001** |
| **Pain score at worst cases** | 7.08 (2.29) | 6.9 (2.35) | 6.86 (2.29) | 6.55 (2.43) | **<0.001** |
| **Days of bleeding** | 5.84 (2.6) | 5.61 (1.7) | 5.68 (1.64) | 5.64 (1.97) | **0.033** |
