# Supplementary material for: Effect of COVID‐19 vaccine on menstrual experience among females in six Arab countries: A cross sectional study
Source: Influenza Other Respir Viruses. 2022 Dec 28;17(1):e13088. doi: 10.1111/irv.13088 (PMC9835440; doi:10.1111/irv.13088)
Supplement: Supplementary file 3 — Supporting Information S3. Effect of demographic characteristics on menstrual experience (a binary logistic regression) [file IRV-17-0-s001.docx]

**Supporting Information S3: Effect of demographic characteristics on menstrual experience (a binary logistic regression)**

| **Covariates included in the first model: country, residency, education, work, smoking, stressful conditions, entertainment sports, covid19 infection, BMI, age, in addition to receiving covid19 vaccine.** | | | |
| --- | --- | --- | --- |
| Factors | Odds ratio (vaccinated to un-vaccinated) | Sig | CI |
| Pelvic pain | 1.257 | 0.004 | (1.076-1.468) |
| Back pain | 1.392 | 0.000 | (1.202-1.611) |
| Thigh pain | 1.11 | 0.086 | (0.985-1.254) |
| Nausea | 1.13 | 0.043 | (1.004-1.273) |
| General weakness | 1.267 | 0.011 | (1.057-1.518) |
| Menstrual pain | 1.351 | 0.000 | (1.154-1.583) |
| Menstrual pain prevalence | 0.744 | 0.000 | (0.637-0.869) |
| Analgesics for menstrual pain (without prescription) | 1.248 | 0.001 | (1.101-1.415) |
| Bowel movement more than usual | 1.888 | 0.001 | (1.289-2.767) |
| Stool more liquid than usual | 1.479 | 0.015 | (1.080-2.024) |
| **Covariates included in the second model: country, residency, education, work in addition to receiving covid-19 vaccine** | | | |
| Factors | Odds ratio (Vaccinated to un-vaccinated) | Sig | CI |
| Pelvic pain | 1.26 | 0.003 | (1.18-1.574) |
| Back pain | 1.363 | 0.000 | (1.202-1.611) |
| Thigh pain | 1.113 | 0.075 | (0.989-1.253) |
| Nausea | 1.131 | 0.038 | (1.007-1.271) |
| General weakness | 1.265 | 0.010 | (1.059-1.511) |
| Menstrual pain | 1.355 | 0.000 | (1.160-1.582) |
| Menstrual pain prevalence | 0.752 | 0.000 | (0.645-0.876) |
| Analgesics for menstrual pain (without prescription) | 1.206 | 0.003 | (1.066-1.364) |
| Bowel movement more than usual | 1.917 | 0.001 | (1.312-2.799) |
| Stool more liquid than usual | 1.473 | 0.015 | (1.079-2.010) |
| **Covariates included in the third model: smoking, stressful conditions, entertainment sports, covid19 infection, BMI, age, in addition to receiving covid19 vaccine.** | | | |
| Factors | odds ratios (Vaccinated to un-vaccinated) | Sig | CI |
| Pelvic pain | 1.248 | 0.005 | (1.071-1.454) |
| Back pain | 1.409 | 0.000 | (1.220-1.628) |
| Thigh pain | 1.136 | 0.036 | (1.008-1.279) |
| Nausea | 1.139 | 0.029 | (1.013-1.281) |
| General weakness | 1.298 | 0.004 | (1.086-1.552) |
| Menstrual pain | 1.319 | 0.000 | (1.129-1.541) |
| Menstrual pain prevalence | 0.740 | 0.000 | (0.635-0.863) |
| Analgesics for menstrual pain (without prescription) | 1.308 | 0.000 | (1.156-1.480) |
| Bowel movement more than usual | 1.817 | 0.002 | (1.247-2.649) |
| Stool more liquid than usual | 1.490 | 0.012 | (1.094-2.031) |
